# Deciphering mechanisms of action of ACE inhibitors in neurodegeneration using *Drosophila* models of Alzheimer’s disease

**DOI:** 10.3389/fnins.2023.1166973

**Published:** 2023-04-11

**Authors:** Judy Ghalayini, Gabrielle L. Boulianne

**Affiliations:** ^1^Program in Developmental and Stem Cell Biology, Peter Gilgin Center for Research and Learning, The Hospital for Sick Children, Toronto, ON, Canada; ^2^Department of Molecular Genetics, University of Toronto, Toronto, ON, Canada

**Keywords:** renin angiotensin system, Alzheimer’s disease, neurodegeneration, *Drosophila*, amyloid, angiotensin converting enzyme

## Abstract

Alzheimer’s disease (AD) is a devastating neurodegenerative disorder for which there is no cure. Recently, several studies have reported a significant reduction in the incidence and progression of dementia among some patients receiving antihypertensive medications such as angiotensin-converting enzyme inhibitors (ACE-Is) and angiotensin receptor blockers (ARBs). Why these drugs are beneficial in some AD patients and not others is unclear although it has been shown to be independent of their role in regulating blood pressure. Given the enormous and immediate potential of ACE-Is and ARBs for AD therapeutics it is imperative that we understand how they function. Recently, studies have shown that ACE-Is and ARBs, which target the renin angiotensin system in mammals, are also effective in suppressing neuronal cell death and memory defects in *Drosophila* models of AD despite the fact that this pathway is not conserved in flies. This suggests that the beneficial effects of these drugs may be mediated by distinct and as yet, identified mechanisms. Here, we discuss how the short lifespan and ease of genetic manipulations available in *Drosophila* provide us with a unique and unparalleled opportunity to rapidly identify the targets of ACE-Is and ARBs and evaluate their therapeutic effectiveness in robust models of AD.

## Introduction

AD is a devastating neurodegenerative disorder that accounts for 70–80% of dementia cases worldwide (Barker et al., 2002). Dementia is a general term used to describe symptoms associated with a decline in cognitive functions including memory, thinking and social abilities that are distinguishable from normal ageing. Currently, around 55 million people have dementia worldwide and as the aging population continues to grow, this number is estimated to triple to 150 million by 2050 (Nichols et al., 2022). In its initial stages, AD is characterized by subtle changes in cognition. However, as the disease progresses, individuals present more severe symptoms including extreme memory loss, impaired spatial and temporal orientation, language disturbances, behavioral changes and motor deficits (Castellani et al., 2010). Ultimately AD renders patients unable to carry out simple day-to-day activities. These symptoms arise from extreme neuronal deterioration mainly in areas of the brain responsible for cognition such as the hippocampus and cortex (Castellani et al., 2010; Holtzman et al., 2011).

AD was first characterized over a century ago, by psychiatrist and neuropathologist Alois Alzheimer who described a 51-year-old patient with memory and language deficits as well as severe disorientation and hallucinations (Graeber et al., 1997; Goedert and Ghetti, 2007). Although these symptoms matched the definition of what was then called dementia, it was atypical for them to be present in someone so young. A post-mortem autopsy revealed various abnormalities in the patient’s brain including extensive atrophy of the cerebral cortex and abnormal protein deposits inside and between nerve cells (Graeber et al., 1997; Goedert and Ghetti, 2007). These protein deposits, known as amyloid plaques and neurofibrillary tangles (NFTs), soon became the pathological hallmarks of AD.

Amyloid plaques in the extracellular matrix of brain tissue are composed of amyloid-β (Aβ) peptides (Glenner and Wong, 1984). These peptides are derived from a precursor transmembrane protein known as APP, found mainly in neurons (Kang et al., 1987; van der Kant and Goldstein, 2015). APP undergoes sequential processing by enzyme complexes, β-secretase and γ-secretase, to produce a C-terminal fragment (C99) followed by an Aβ peptide, respectively (Vassar et al., 1999; Haapasalo and Kovacs, 2011; van der Kant and Goldstein, 2015). C99 may be cleaved at different sites by γ-secretase thus, amyloid-β peptides vary in size ranging from 38 to 43 residues, with Aβ40 and Aβ42 being the most prominent species (Takami et al., 2009). Aβ40 and Aβ42 only differ by two residues however, Aβ42 is more prone to aggregation and is found enriched in amyloid plaques (Miller et al., 1993; Iwatsubo et al., 1994; Mak et al., 1994). Furthermore, Aβ42 has a much higher level of neurotoxicity compared to Aβ40. The ratio of Aβ42/Aβ40 is often used as a reference to AD pathogenesis (Kuperstein et al., 2010). However, Aβ levels and deposition appear to plateau at the onset of clinical symptoms and correlate poorly with the degree of cognitive impairment in the dementia phases of AD (Masters et al., 2015). In contrast, measurements of biomarkers for neurofibrillary tangles (NFTs) strongly correlate with disease progression and severity at the onset of clinical manifestation (Masters et al., 2015).

NFTs are intraneuronal aggregates mainly composed of hyperphosphorylated Tau, a microtubule associated protein that interacts with tubulin to support its assembly into microtubules and to stabilize its structure (Barbier et al., 2019). Tau is also known to promote neurite outgrowth and axonal transport (Barbier et al., 2019). However, in AD, tau proteins are hyperphosphorylated, which causes them to assemble into filamentous bundles (Busche and Hyman, 2020). As a result, the structural integrity of microtubules is damaged, impairing axonal transport and neurite development, which leads to neuronal cell death (Busche and Hyman, 2020). Accordingly, NFTs and phosphorylated tau levels correlate strongly with the degree of neurodegeneration in AD patients and are predictive of disease severity (Masters et al., 2015).

The relationship between Aβ and NFTs is complex. Findings show high Aβ load - either as plaques or non-fibrillar, soluble, oligomeric forms, precede NFT formation and suggest that Aβ initiates AD in part by acting on pathophysiological mechanisms that lead to tau-hyperphosphorylation and aggregation into NFTs (Busche and Hyman, 2020). Consistent with this model, animal studies have demonstrated that Aβ peptides promote tau-hyperphosphorylation resulting in its aggregation into NFTs (Götz et al., 2001; Gomes et al., 2019). This concept is consistent with the amyloid-cascade hypothesis whereby Aβ is the primary initiator of AD (described below).

An understanding of how Aβ peptides lead to senile plaque formation was revealed through familial genetic studies of AD, which identified dominant mutations in APP and presenilin 1 and presenilin 2; the core catalytic components of γ-secretase (Holtzman et al., 2011; van der Kant and Goldstein, 2015). These mutations were shown to profoundly alter APP metabolism, increasing and favoring the production of aggregation prone Aβ species such as Aβ42 (Holtzman et al., 2011; van der Kant and Goldstein, 2015). Ultimately these studies led to the development of the amyloid cascade hypothesis of AD pathogenesis (Hardy and Selkoe, 2002). This long-standing hypothesis states that neurotoxic forms of β-amyloid peptides initiate AD pathology and precede all other disease hallmarks. However, familial AD (FAD), also known as early-onset AD (EOAD), accounts for only 1–5% of all cases (Holtzman et al., 2011). Most cases of AD fall under the “sporadic” late-onset form (LOAD), which lacks clearly defined genetic factors. However, genome-wide association studies (GWAS) have identified certain genetic risk factors including the apolipoprotein E gene (apoE) (Bertram and Tanzi, 2009). Individuals carrying the apoE e4 allele have up to a 12-fold increased risk of developing the disease (Bertram and Tanzi, 2009). As more genetic risk factors are discovered, it becomes increasingly clear that AD is a complex and multifactorial disorder.

Neuroinflammation has also emerged as a significant factor in the pathogenesis of AD, alongside amyloid plaques and NFT (Newcombe et al., 2018). Neuroinflammation refers to an inflammatory response that occurs within the CNS and involves the production of cytokines, chemokines, reactive oxygen species and secondary messengers. Microglial cells are the primary players in neuroinflammation and act as the resident macrophages of the CNS, serving several roles including acting as a first line of immune defence against brain injury or infection (Calsolaro and Edison, 2016). However, it has been suggested that the activation of microglia and the subsequent release of pro-inflammatory factors can cause significant neuronal damage. Although microglial activation can be protective within the CNS, if the stimulus for activation is not resolved, chronic inflammation can develop and contribute to neuronal dysfunction, injury and loss (Calsolaro and Edison, 2016; Fakhoury, 2018). Post-mortem studies of AD patient brains have revealed activated microglia co-localized with amyloid plaques. Amyloid peptides, fibrils and APP have also been shown to activate microglia, triggering an inflammatory response and the release of neurotoxic cytokines (Calsolaro and Edison, 2016; Fakhoury, 2018). PET studies have demonstrated a correlation between microglial activation and amyloid load in AD patients. Moreover, neuroinflammation has been detected before the onset of dementia, suggesting its early occurrence in AD pathology. Finally, GWAS studies have identified a relationship between components of the innate immune system and the incidence of sporadic AD, further supporting a link between the immune system and AD (Newcombe et al., 2018).

Despite distinct etiologies of sporadic and familial forms of AD, both exhibit comparable clinical manifestations, including rates of disease progression and similar biomarker profiles. As a result, genetic factors known in FAD are commonly employed to model the disease in research aimed at understanding its underlying causes. Moreover, these models are widely utilized in preclinical studies to evaluate the effectiveness of potential therapeutics.

Recently, there has been a significant advancement in the treatment of AD with the approval of two monoclonal antibodies, aducanumab and lecanemab, as potential therapies for AD (Tampi et al., 2021; Reardon, 2023). These drugs are designed to target and clear β-amyloid plaques. However, while clinical studies have demonstrated their efficacy in reducing Aβ levels, their impact on cognitive improvement has been limited (Tampi et al., 2021; Reardon, 2023). This suggests that targeting Aβ and tau alone may not be sufficient to effectively treat AD and that a more comprehensive approach that targets multiple pathways may be more effective. Therefore, ongoing research to develop therapies that target other aspects of AD is crucial. One promising avenue of research revolves around the potential benefits of drugs that target the renin angiotensin system (RAS).

### RAS in AD

Systemic RAS (or peripheral RAS) is a hormonal system that plays a critical role in regulating blood pressure and fluid balance in the body (Yim and Yoo, 2008; Wu et al., 2018). It is made up of several hormones and enzymes, including renin, angiotensinogen, angiotensin (Ang) I, Ang II, and angiotensin converting enzyme (ACE). Renin is produced and released by the kidneys in response to low blood pressure or low blood volume and it acts on angiotensinogen (a pro-peptide produced by the liver) to produce Ang I (Yim and Yoo, 2008; Wu et al., 2018). Ang I is then converted to Ang II by ACE, a zinc- and chloride-dependent metallopeptidase, which is expressed in the lungs and other tissues (Yim and Yoo, 2008; Wu et al., 2018). Ang II is a potent vasoconstrictor, exerting its effects by binding to angiotensin II type 1 receptors (AT1Rs), while binding to angiotensin II type 2 receptors (AT2Rs) induces vasodilation (Yim and Yoo, 2008; Wu et al., 2018). Ongoing research of the RAS has led to the discovery of additional components, such as ACE2, a homolog of ACE. ACE2 cleaves Ang I or Ang II into the heptapeptide angiotensin 1–7 (Ang 1–7). This peptide was later found to bind to the Mas receptor (MasR), resulting in vasodilation (Yim and Yoo, 2008; Wu et al., 2018).

A pivotal discovery relating to the RAS, was the development of captopril, the first ACE inhibitor (ACE-I) drug in 1975 (Zheng et al., 2022). Captopril selectively targets ACE by binding to its active site and preventing the formation of Ang II thereby inhibiting the RAS. It marked a significant milestone in the treatment of hypertension paving the way for the development of additional ACE-Is with improved activity and bioavailability (Zheng et al., 2022). In addition, a new class of drugs known as angiotensin receptor blockers (ARBs), which selectively inhibit Ang II by competitive antagonism of the AT1Rs, were also developed (Barreras and Gurk-Turner, 2003). These drugs have proven to be clinically relevant in the treatment of various cardiovascular conditions, including hypertension, heart failure, diabetic nephropathy and continue to be an important therapeutic option for patients today (Barreras and Gurk-Turner, 2003; Zheng et al., 2022).

Well after its discovery, it became evident that the RAS system is also expressed in numerous organs including the brain (now referred to as local RAS) and possesses additional functionalities revealing its degree of complexity (Paul et al., 2006). Local synthesis of cerebral RAS is necessary due to the blood–brain barrier (BBB) preventing peripheral RAS components from accessing most regions of the brain (Jackson et al., 2018). Astrocytes are the primary source of angiotensinogen, which is constitutively secreted and cleaved into various neuroactive peptides (Jackson et al., 2018). As previously described and illustrated in w, renin converts angiotensinogen to Ang I, which is further processed by ACE to produce Ang II. Ang II is the main effector protein that binds to AT1R or AT2R. Ang II can also be further processed into Ang IV by aminopeptidases (AP-A and AP-N), which binds to AT4R (Jackson et al., 2018). Alternatively, ACE2 converts Ang II to Ang 1–7, which binds to MasRs. Ang 1–7 can also be produced through first processing of Ang I by ACE2 to produce Ang 1–9 and then by ACE (Jackson et al., 2018). AT1R and AT2R are present in neurons, astrocytes, oligodendrocytes and microglia of the cortex, hippocampus and basal ganglia both on the cell surface and intracellularly at mitochondrial and nuclear levels allowing for the regulation of oxidative stress, transcription and trafficking of receptor types (Jackson et al., 2018). Activation of AT1R has been associated with deleterious effects such as promoting neuroinflammation, oxidative stress and neuronal cell death (Jackson et al., 2018; Cosarderelioglu et al., 2020). In contrast, AT2R appears to be neuroprotective, counteracting AT1R’s effects by inhibiting neuroinflammation, reducing oxidative stress and influencing neuronal regeneration. Of note, while Ang II can bind both receptors, ACE upregulation specifically leads to increased AT1R activation (Jackson et al., 2018). MasR also located in these brain regions are expressed by neurons, astrocytes and microglia, and similar to AT2R, have both antioxidant and anti-inflammatory properties and promote cell survival (Jackson et al., 2018). The expression of AT4R is restricted to neurons localized in the cortex, hippocampus and basal ganglia, where it is believed to induce LTP and mediate learning and memory consolidation (Jackson et al., 2018; Cosarderelioglu et al., 2020). The interplay between the receptors and enzymes of RAS in the brain has been suggested to work synergistically and is thought to be crucial in maintaining cognitive balance in a healthy brain. Accordingly, misregulation of RAS has been implicated in pathologies underlying neurodegenerative diseases including AD (Cosarderelioglu et al., 2020; Gouveia et al., 2022). Changes in RAS components have been documented in brains of AD patients compared to control individuals (Savaskan et al., 2001). For example, studies have found increased levels of ACE in the hippocampus, frontal cortex and caudate nucleus of AD patients and its activity is increased and correlates positively with parenchymal Aβ load (Arregui et al., 1982; Miners et al., 2008; MacLachlan et al., 2022). While the expression of ACE appears to be upregulated in AD, the opposite has been reported for ACE2. Researchers observed a significant reduction in ACE2 activity in the mid-frontal cortex of AD patients and this reduction was inversely correlated with total β-amyloid and tau load as well as ACE activity (Kehoe et al., 2016). Similarly, a systematic analysis of ACE2 protein expression in different brain regions revealed a downregulation in the basal nucleus, hippocampus and entorhinal cortex, middle frontal gyrus, visual cortex and amygdala of AD patient brains (Cui et al., 2021). However, contrary to these findings, Ding et al. (2021) reported higher ACE2 protein expression levels in hippocampal tissues of AD patients compared to control subjects. Although, in a more recent study that evaluated ACE and ACE2 protein expression and enzyme activity in the frontal and temporal cortex in early AD stages, authors report that both ACE and ACE2 protein level are unchanged and that only ACE enzyme activity was elevated (MacLachlan et al., 2022). ACE and ACE2 act on different axes of the RAS having either neurotoxic or neuroprotective properties, respectively and an imbalance between these axes may play a role in AD pathogenesis. Taken together, these discrepancies suggest the need for further analysis.

Genetic studies have also implicated ACE as a probable risk factor for AD (Alvarez et al., 1999; Elkins et al., 2004). Recent AD GWAS meta-analyses identified common genetic variants in the *Ace* locus outside of exonic regions, which are associated with an increased risk of AD (Marioni et al., 2018; Kunkle et al., 2019). In the past, genetic studies of *Ace* and AD have focused primarily on a common insertion/deletion (I/D) variant that influences ACE serum levels. Individuals with the D/D haplotype have higher serum ACE levels than those with I/I haplotype (Elkins et al., 2004). Most studies, Kehoe et al. (1999), Narain (2000), Kölsch et al. (2005), and Lehmann et al. (2005) although at times contradictory (Chou et al., 2016), have indicated that individuals carrying an insertion allele are at higher risk of AD than those with the D/D haplotype. It is important to note that ACE serum levels do not reflect ACE enzymatic activity levels. In a more recent genetic study, Cuddy et al. (2020) performed whole genome sequencing to identify rare coding variants in the *Ace* gene associated with AD. They selected one variant (R1297Q) for functional analysis to gain a better understanding of the role that ACE plays in AD. This was achieved by generating knock-in (KI) mice that harbored the cognate mutation, R1297Q, in the murine *Ace* gene. The authors report that while the mutation had no effect on blood pressure and cerebrovasculature, it did result in increased levels of neuronal ACE protein and activity, memory impairment, neuroinflammation and hippocampal neurodegeneration. Moreover, these reported phenotypes were exacerbated in an AD mouse model of amyloidosis. These findings strongly suggest that increased ACE activity is associated with AD pathogenesis.

Following this apparent genetic link between RAS and AD, epidemiological and clinical studies were performed to examine the effects of RAS targeting drugs including ACE-Is and ARBs on the incidence of AD. A retrospective study conducted by Barthold et al. (2018) assessed the risk of developing AD in patients being treated with either RAS targeting or non-RAS anti-hypertensive medication. The study findings indicated that RAS-acting drugs were more effective in reducing the risk of AD development compared to non-RAS acting drugs. The study also compared the effects between ARBs and ACE-Is and found that ARBs demonstrated superior preventative efficacy against AD than ACE-Is. These findings are in line with a previous prospective cohort study conducted by Li et al. (2010) that reported reduced incidence and progression of AD in participants taking ARBs compared to those taking other cardiovascular drugs and a nested case–control analysis following AD patients who were prescribed different anti-hypertensive drugs including ACE-Is and ARBs (Davies et al., 2011). Specifically, this study found a 53% decrease in AD incidence for ARB use and a 24% decrease in AD incidence from ACE-I use (Davies et al., 2011). Furthermore, ACE-Is and ARBs have also been evaluated for their ability to reduce the rate of cognitive decline and improve cognitive performance in AD patients. From a cross-sectional and retrospective cohort study of an elderly population, Hajjar et al. (2005) reported patients taking ARBs had improved cognitive performances while those taking ACE-Is presented a lower rate of cognitive decline. A follow up double-blind randomized clinical trial reported similar findings whereby the ARB, candesartan, was associated with improvement in cognition and outperformed the ACE-I, lisinopril (Hajjar et al., 2012). While studies continue to support the potential benefits of ARBs in AD (Ouk et al., 2021; Deng et al., 2022), a recent clinical study evaluating the effect of 12-month losartan (an ARB) treatment on brain atrophy in patients diagnosed with mild-to-moderate AD reported no significant reduction in brain volume loss (Kehoe et al., 2021). These findings suggest that further studies regarding ARB treatment duration and time of treatment are needed to determine when potential benefits of ARB use may arise. Regarding ACE-Is, their potential benefits for AD are not as clear. Even though a prospective cohort study by Soto et al. (2013) showed a slower rate of cognitive decline in older adults taking ACE-Is, it appears that ACE-Is as a pharmacological class do not reduce the risk of developing dementia or improve cognition in AD (Sink et al., 2009; Solfrizzi et al., 2013; O’Caoimh et al., 2014). However, a closer examination of subgroups of ACE-Is, such as those that can penetrate the BBB vs. those that cannot, imply potential beneficial effects may arise exclusively from drugs that can penetrate the BBB (Ellul et al., 2006; Gao et al., 2013; O’Caoimh et al., 2014). Indeed, in an observational study, O’Caoimh et al. (2014) found a decrease in the rate of cognitive decline in patients with mild to moderate AD receiving BBB-penetrating ACE-Is compared to those on no drug. Finally, it is important to note that the beneficial effects of ACE-Is and ARBs remain the same even after studies adjusted for blood pressure or hypertension suggesting that the beneficial effects are independent of blood pressure or hypertension regulation (Hajjar et al., 2008). While the precise mechanisms by which ACE-Is and ARBs exert their effects in AD remain unclear, their promise as potential therapeutics has inspired researchers to elucidate their mechanisms of action utilizing *in vivo* model systems.

Several studies have now examined the effects of ARBs on cognition animal models, as shown in Table 1. While the majority of studies have demonstrated that ARBs can improve learning and memory in AD mouse models (Wang et al., 2007; Takeda et al., 2009; Ongali et al., 2014; Royea et al., 2017; Torika et al., 2017) others have failed to demonstrate any or have shown limited beneficial effects (Papadopoulos et al., 2016; Trigiani et al., 2018). Nonetheless, improvements in memory performance and retrieval, spatial learning, and prevention of cognitive deficits have been documented through the use of different ARBs such as losartan, olmesartan, and telmisartan (Wang et al., 2007; Takeda et al., 2009; Ongali et al., 2014; Royea et al., 2017; Torika et al., 2017). These beneficial effects are suggested to result, in part, by the ability of ARBs to reduce β-amyloid load, neuroinflammation and oxidative stress in the brain (Wang et al., 2007; Takeda et al., 2009; Danielyan et al., 2010; Torika et al., 2017, 2018) all of which are neuropathological hallmarks of AD (Krstic and Knuesel, 2012; Cassidy et al., 2020). With the increasing number of studies in this field, it is evident that ARBs have strong therapeutic potential and efforts into revealing their mechanism of action are underway.

**Table 1:** Summary of effects of ARBs in mouse models of AD including Aβ levels and deposition, neuroinflammation and oxidative stress and, cognitive deficits.

Mouse model	Drug (*brain penetrant)	Treatment duration and age	Administration route	Dose	Results	References
APP/PSEN1 mice (APP^Swedish^/PSEN1^1dE9^)	Losartan*	2 months (every other day), starting at 7 months old	Intranasal administration	10 mg/kg	↓ Aβ plaques; ↓ Inflammation	Danielyan et al. (2010)
APP mice (APP^Swedish/Indiana^)	Losartan*	3 months, starting at 4 months old	Drinking water	10 mg/kg/d	↑ Learning and memory; ↓ Inflammation	Royea et al. (2017)
A/T mice (APP^Swedish/Indiana^, active TGF-β1 form)	Losartan*	3 months, starting at 3 months old	Drinking water	10 mg/kg/d	No improvement in learning or memory; no effect on Aβ levels and plaques; no effect on increased inflammatory response	Papadopoulos et al. (2016)
APP mice (APP^Swedish/Indiana^)	Losartan*	3 months, starting at 15 months of age	Drinking water	10 mg/kg/d	↑ Memory; no effect on learning deficit; no effect on Aβ plaques; ↓ oxidative stress	Ongali et al. (2014)
5XFAD Tg mice (APP^Swedish/Florida/London^ /PSEN1^M146L, L286V^)	Candesartan*	8 weeks, starting at 2 months of age	Intranasally	1 mg/kg/d	↓ Aβ plaques; ↓ Inflammation	Torika et al. (2018)
APP mice (APP^Swedish/Indiana^)	Candesartan *	5 months, starting at 3–4 months of age	Drinking water	10 mg/kg/d	Limited cognitive improvement; no effect on Aβ plaques or oxidative stress; ↓ Inflammation	Trigiani et al. (2018)
APP23 mice	Olmesartan	4/5 weeks, starting at 12/13 weeks of age	Oral administration	1 mg/kg/d	↑ Memory and learning; ↓ oxidative stress; no effect on Aβ levels	Takeda et al. (2009)
5XFAD Tg mice (APP^Swedish/Florida/London^ /PSEN1^M146L, L286V^)	Telmisartan*	5 months, starting at 8 weeks of age	Intranasal	1 mg/kg/d	↓ Aβ plaques;↓ Inflammation;↑ learning	Torika et al. (2017)
Tg2576 mice (APP^Swedish^)	Valsartan*	5 months, starting at 6 months of age	Drinking water	10 and 40 mg/kg/d	↑ Memory andlearning; ↓ Aβ plaques	Wang et al. (2007)

Drugs marked with (*) indicate BBB penetrance.

However, as summarized in Table 2, the effects of ACE-Is in AD are more ambiguous. Some studies suggest that ACE-Is may be detrimental as they led to an increase in Aβ accumulation in AD mice (Zou et al., 2007; Liu et al., 2019). For example, Liu et al. (2019) reported that treating Tg2576 AD mice with captopril, an ACE-I, for 11 months resulted in increased levels of Aβ42 and β-amyloid plaque deposition in the hippocampus and neocortex. In contrast, other studies have shown that captopril treatment reduced Aβ burden (AbdAlla et al., 2013; Torika et al., 2016; Asraf et al., 2018). For example, AbdAlla et al. (2013) demonstrated that treating Tg2576 AD mice with captopril for 6 months led to reduced amyloidogenic processing of full-length APP resulting in slower accumulation of Aβ in the hippocampus. However, other studies reported no effect of ACE inhibition on Aβ (Hemming et al., 2007; Dong et al., 2011). These discrepancies make it difficult to conclude whether ACE inhibition may be beneficial in AD. However, an evaluation of the differences in a number of factors across these studies including, method of administration, drug dose and treatment duration may help explain the contrasting results. For instance, Hemming et al. (2007) evaluated the effect of different concentrations of captopril delivered by oral administration in AD mice over 28 days on its ability to inhibit ACE activity in the brain. They found that only high concentrations of captopril, approximately 10 times relative to the highest amount used in therapeutic doses in humans, led to a significant but modest reduction in ACE activity and there was no change in either cerebral Aβ levels or deposition. In a related study by Liu et al. (2019), captopril was administered orally at similar concentrations resulting in a significant reduction in systolic and diastolic blood pressure likely due to inhibition of peripheral ACE. However, no data was provided as to whether there was a significant effect on inhibiting brain ACE that could account for the increased levels of Aβ42 observed by the authors as a result of captopril treatment. In contrast, studies whereby captopril was administered intranasally reported reduced Aβ42 load in hippocampal and cortical areas (Torika et al., 2016; Asraf et al., 2018). It is worth noting that treatment duration also impacts the outcome of captopril treatment. Torika et al. (2016) found that when AD mice were treated with captopril for 3.5 weeks vs. 7 months, no changes in β-amyloid load were observed. However, this was not the case when they administered perindopril, another ACE-I, for a short duration suggesting that the type of ACE-I used could contribute to discrepancies in the literature. Finally, in addition to altering Aβ levels, ACE-Is appear to alter immune responses in brains of AD mice similar to that observed using ARBs (Dong et al., 2011; Torika et al., 2016; Asraf et al., 2018). For example, studies showed that ACE inhibition reduced the level of CD11b, a marker of activated microglial and reactive oxygen species (ROS) suggesting an overall reduction in inflammation (AbdAlla et al., 2013; Torika et al., 2016; Asraf et al., 2018). Altogether, these studies suggest that while ARBs and ACE-Is may have beneficial effects in AD animal models further studies are needed to decipher both the method of delivery and their mechanism of action.

**Table 2:** Summary of effects of ACE-Is in mouse models of AD including Aβ levels and deposition, neuroinflammation and oxidative stress and, cognitive deficits.

Mouse model	Drug (*brain penetrant)	Treatment duration and Age	Administration route	Dose	Results	Reference
Tg2576 mice (APP^Swedish^)	Captopril*	11 months, starting at 6 months old	Oral administration	30 mg/kg/d	↑Aβ levels and plaques	Zou et al. (2007)
Tg2576 mice (APP^Swedish^)	Captopril*	11 months, starting at 6 months old	Oral administration	30 mg/kg/d	↑Aβ levels and plaques	Liu et al. (2019)
3xTg-AD mice (APP^Swedish^/PSEN1^M146V^/Tau^P301L^) J20 mice (APP^Swedish/Indiana^)	Captopril*	1 months, starting at 16 months old	Drinking water	2 g/l	No effect on Aβ levels and plaques	Hemming et al. (2007)
PS2APP Tg mice (APP^Swedish^/PSEN2^N141I^)	Perindopril*	1 months, starting at 3 months old	Oral administration	1 mg/kg/d	↑ Memory; No effect on Aβ levels; ↓ Inflammation	Dong et al. (2011)
Tg2576 mice (APP^Swedish^)	Captopril*	6 months, starting at 12 months old	Drinking water	20 mg/kg/d or 25 mg/kg/d	↓ Aβ plaques; ↓ oxidative stress	AbdAlla et al. (2013)
5XFAD Tg mice (APP^Swedish/Florida/London^ /PSEN1^M146L, L286V^)	Captopril*	2 months, starting at 8 weeks old	Intranasal administration	5 mg/kg/d	↓ Aβ plaques; ↓ Inflammation	Asraf et al. (2018)
5XFAD Tg mice (APP^Swedish/Florida/London^ /PSEN1^M146L, L286V^)	Captopril*	3.5 weeks, starting at 3 months old	Intranasal administration	5 mg/kg/d	No effect on Aβ plaques; ↓ Inflammation	Torika et al. (2016)
5XFAD Tg mice (APP^Swedish/Florida/London^ /PSEN1^M146L, L286V^)	Perindopril*	3.5 weeks, starting at 3 months old	Intranasal administration	1 mg/kg/d	↓ Aβ plaques; ↓ Inflammation	Torika et al. (2016)
5XFAD Tg mice (APP^Swedish/Florida/London^ /PSEN1^M146L, L286V^)	Captopril*	7 months, starting at 2 months old	Intranasal administration	5 mg/kg/d	↓ Aβ plaques	Torika et al. (2016)

Drugs marked with (*) indicate BBB penetrance.

### Drosophila as a model for Alzheimer’s disease

*Drosophila* has proven to be an excellent model system to study neurodegenerative diseases. The short life span of flies coupled with powerful genetic approaches has made it possible to generate models of disease that faithfully recapitulate many features observed in patients, including age-dependent neurodegeneration and progressive defects in synaptic plasticity and memory. Once a model has been generated it can also be used to perform genetic screens to identify modifiers of known disease-causing genes or drug screens to identify and evaluate novel therapies. Many of the genes associated with neurodegeneration are also conserved in *Drosophila* including those implicated in AD. For example, *Drosophila* possess a homolog of APP known as APP-like or APPL (Luo et al., 1992). Flies deficient in this gene exhibit a behavioral defect that can be partially rescued by expressing a human APP transgene suggesting functional homology between APP and APPL (Luo et al., 1992). However, APPL lacks the amyloidogenic Aβ peptide sequence at the C- terminus found in human APP and does not appear to be processed *in vivo* as is human APP (Prüßing et al., 2013). Similarly, while Presenilin and the γ-secretase complex are well conserved (Periz and Fortini, 2004) there is no clear homolog of β-secretase in flies (Prüßing et al., 2013).

Several transgenic fly lines based on the expression of human and/or *Drosophila* AD-related genes have been generated and are readily available to study disease processes. One such model is based on co-expression of human β-secretase along with human APP and fly presenilin, both of which possess FAD-linked mutations (Greeve et al., 2004). Co-expression of all three transgenes in the fly eye led to β-amyloid plaque formation and age-dependent neurodegeneration (Greeve et al., 2004) demonstrating that *Drosophila* could be used as a model for AD. Soon after, additional models were generated, whereby transgenic flies expressed different Aβ transgenes in specific tissues. The transgenes varied in several ways, including whether or not they possessed a signal sequence to allow for expression outside cells, the number of Aβ42 copies expressed in tandem, and whether they contained an FAD mutation (Prüßing et al., 2013). All exhibited similar phenotypic defects when expressed in neuronal tissue, including plaque formation, neurodegeneration, as well as motor and cognitive defects (Prüßing et al., 2013). The main difference between the models was the severity of the phenotypes, which often correlated with the levels of Aβ42 expression and the degree of protein aggregation. Transgenic flies that express Tau in neuronal tissue are also available and similarly show robust phenotypes reminiscent of those observed in AD (Prüßing et al., 2013). Functional genomic studies using various AD fly models have also facilitated our understanding of the role of cellular mechanisms including inflammation, oxidative stress, mitochondrial dysfunction and apoptosis in AD pathogenesis (reviewed by Jeon et al., 2020). Finally, the ability to perform large-scale genetic screens together with the availability of RNA interference for all genes annotated in the fly genome, make it possible to identify novel modifiers of Aβ42 and Tau (Jeon et al., 2020) that not only provide insight into the molecular and cellular pathways implicated in AD but also potential novel therapeutic targets for this devastating disease.

### Ace and Drosophila AD models

The RAS has been well studied in humans and many mammalian model organisms due to its intricate role in regulating blood pressure. However, components of the RAS have also been found in non-mammalian organisms that lack a closed circulatory system (Fournier et al., 2012). ACE homologs have been identified in several invertebrate organisms, including *Drosophila*. There are six ACE-like factors in *Drosophila*, including Ance, Ance-2,-3,-4,-5, and Acer (Cornell et al., 1995; Taylor et al., 1996; Houard et al., 1998). Of these, only Acer and Ance are believed to be active zinc metallopeptidases as they possess an intact conserved active site motif (HExxH) (Coates et al., 2000). Their catalytic activity was demonstrated through biochemical assays that showed their ability to hydrolyze an ACE synthetic substrate, Hip-His-Leu, which mimics the C-terminal sequence of Ang I, to a similar degree as mammalian ACE (Houard et al., 1998; Coates et al., 2000).

ACE-Is have also been shown to be effective in inhibiting Acer and Ance catalytic activity although both enzymes structurally differ from their mammalian counterparts (Houard et al., 1998; Coates et al., 2000; Bingham et al., 2006; Akif et al., 2012). In mammals, the ACE gene is subjected to alternative splicing that gives rise to two distinct enzymes (Hubert et al., 1991). Somatic ACE (sACE), is widely distributed and contains two active sites (N and C-terminal) while germinal ACE (gACE), is expressed exclusively in the testes and only possesses the second active site (C-terminal) (Hubert et al., 1991; Shen et al., 2008). *Drosophila* Acer and Ance only possess a single catalytic domain (Houard et al., 1998; Coates et al., 2000). The active site of Acer is similar to the N-terminal active site of somatic ACE, whereas the active site of Ance is similar to the C-terminal active site of somatic ACE. Moreover, Acer and Ance lack a C-terminal transmembrane anchor that is found in mammalian ACE and may be proteolytically cleaved to yield soluble enzymes (Houard et al., 1998; Coates et al., 2000). However, both Acer and Ance possess a signal sequence that leads to their secretion outside the cell (Coates et al., 2000; Rylett et al., 2007; Carhan et al., 2011). A comparison between *Drosophila* and human ACE proteins is illustrated in Figure 2.

The first study to suggest a link between ACE and AD in *Drosophila* was a modifier screen aimed to identify genes that either enhanced or suppressed phenotypes resulting from over-expression of *Psn* followed by a secondary screen to determine if any of the *Psn* modifiers could also suppress phenotypes generated from expression of the truncated form of APP, called C99 (van de Hoef et al., 2009). Numerous candidates that suppressed or enhanced both the Psn and C99 phenotypes were identified, including some that had previously been shown to interact with Psn. More importantly, two ACE-like factors were identified: *Acer* and *Ance-5*. *Acer* and *Ance-5* modified Psn-dependent phenotypes while *Ance-5* also modified the C99-dependent phenotype (van de Hoef et al., 2009). This suggested that ACE-like factors might be involved in regulating Psn function and that further characterization of the interaction between psn, APP, and ACE might aid our understanding of AD pathogenesis and the potential of ACE-Is for AD therapeutic development. However, while there is increasing evidence that ACE-Is may be beneficial in AD patients, the mechanisms behind the beneficial effects of the drugs remain poorly understood. One reason is the inability to disentangle their effects on blood pressure from their direct effects on local RAS. As such, *Drosophila*, which does not have a conserved RAS pathway (Salzet et al., 2001; Fournier et al., 2012), provides a unique model to study the relationship between ACE-Is and AD. Toward this goal, Lee et al. (2020) evaluated the effects of ACE-Is and ARBs in fly lines expressing different AD-related transgenes. Using the GAL4/UAS system, fly lines were generated that expressed the following human transgenes: C99^WT^ and C99^V717I^ (a common mutation of APP found in FAD), and Aβ42 in CNS tissue, including the eye and brain. They showed that all of the flies exhibit age-dependent defects, including neuronal cell death and impaired memory, with the mildest defects observed in C99^WT^ flies and the most severe in Aβ42 flies. Importantly, both the cell death and memory defects observed in C99 and Aβ42 flies were suppressed when flies were fed either captopril (an ACE-I) or losartan (an ARB), as summarized in Table 3. Moreover, measurements of Aβ42 levels and plaques in drug treated flies were similar to untreated flies, suggesting that the observed beneficial effects are independent to changes in Aβ42 pathology. This is consistent with findings from some of the mouse studies mentioned previously. Finally, to confirm that ACE-Is work in *Drosophila* in a similar manner to mammals, Lee et al. (2020) evaluated the effects of an *Acer* null mutant on various transgenic AD-related lines mentioned above. They found that a complete loss of *Acer* recapitulated the effects of captopril demonstrating that captopril exerts its effects by specifically inhibiting Acer.

**Table 3:** Summary of effects of ACE-Is and ARBs in *Drosophila* models of AD including Aβ levels and deposition, oxidative stress and, cognitive deficits.

*Drosophila* model	Drug (*brain penetrant)	Treatment duration and Age	Administration route	Dose	Results	Reference
hAPP, hBACE/+; elav-gal4/+	Lisinopril* (ACE-I)	5–7 days, 1 day old	Mixed in food	1 mM	↑ Learning and memory; ↑ climbing ability; ↓ oxidative stress (decrease H_2_O_2_ in thoracic)	Thomas et al. (2021)
elav-gal4/+; UAS-APP^C99V717I^	Captopril* (ACE-I)	4 weeks, 1 day old	Mixed in food	5 mM	↑ Memory; ↓ cell death; no change in C99 levels	Lee et al. (2020)
elav-gal4/+; UAS-APP^C99V717I^	Losartan* (ARB)	4 weeks, 1 day old	Mixed in food	1 mM	↓ cell death; no change in C99 levels	
elav-gal4/+; UAS-APP^Abeta42.B^	Captopril* (ACE-I)	4 weeks, 1 day old	Mixed in food	5 mM	↑ Memory; ↓ cell death; No effect on Aβ levels and plaques	
elav-gal4/+; UAS-APP^Abeta42.B^	Losartan* (ARB)	4 weeks, 1 day old	Mixed in food	1 mM	↑ Memory; ↓ cell death; No effect on Aβ levels and plaques	

In a similar study, Thomas et al. (2021) aimed to determine whether the administration of lisinopril, an ACE-I, would have beneficial effects in a *Drosophila* model of AD that overexpresses human APP and human β-secretase in CNS tissue. Compared to control flies, these flies displayed deficits in learning and memory as well as in climbing ability, as determined through an aversive phototaxis suppression assay and a negative geotaxis assay, respectively. Upon lisinopril administration, AD flies displayed both improved cognition and climbing ability as well as a significant reduction in oxidative stress levels compared to untreated AD flies, as shown in Table 3.

To date, the mechanisms by which ARBs and ACE-Is function to suppress cell death and memory defects in *Drosophila* remain unknown. The effects of losartan are surprising given that their known target in mammals, AT1R, is not conserved in flies (Fournier et al., 2012). These findings suggest that losartan functions through an unknown and potentially novel target in *Drosophila*. This warrants further studies to help elucidate what its target(s) in flies might be. With regards to ACE-Is, Lee et al. (2020) demonstrated that the ability of captopril to suppress AD-related phenotypes in flies could be recapitulated by a null mutation in *Acer* demonstrating that Acer or a downstream effector of Acer, is the target of captopril. However, as previously mentioned, apart from ACE, RAS substrates do not appear to be conserved in *Drosophila* (Fournier et al., 2012) suggesting the existence of novel targets for ACE that extend beyond its conventional role in the canonical RAS. Such targets can be readily identified and validated in flies using a combination of biochemical and genetic approaches. Characterization of these targets will not only reveal the cellular pathways on which Acer acts in *Drosophila* but may also reveal novel roles for mammalian ACE beyond its canonical role in RAS and lead to the development of additional therapies for AD.

**Figure 1:**
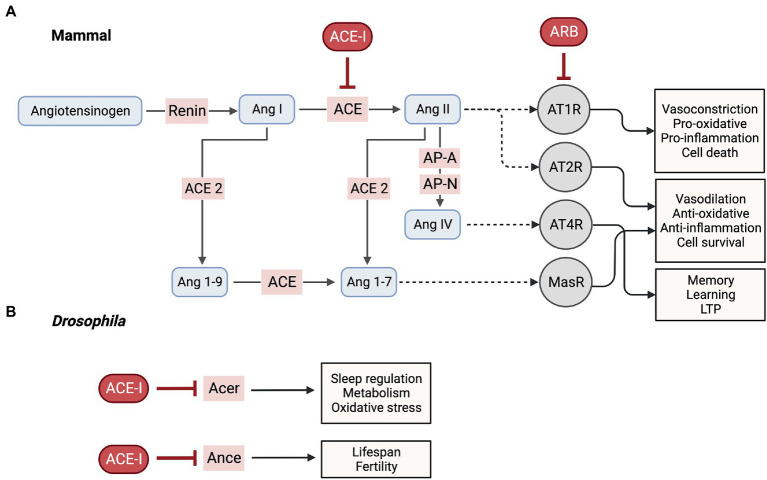
RAS in mammals vs. *Drosophila*. **(A)** Mammalian brain RAS pathways and its inhibitors. ACE, angiotensin converting enzyme; ACE-I, angiotensin converting enzyme inhibitor; Ang, angiotensin; AP-A, aminopeptidase A; AP-N, aminopeptidase N; ARB, angiotensin receptor blocker; AT1R, angiotensin II type 1 receptor; AT2R, angiotensin II type 2 receptor; AT4R, angiotensin 4 receptor; LTP, long-term potentiation; MasR, Mas receptor. **(B)** ACE-I inhibit Acer and Ance, the *Drosophila* ACE homologs. Acer, angiotensin-converting-enzyme related; Ance, angiotensin converting enzyme. (Created with BioRender.com).

Although the molecular targets of Acer in *Drosophila* are unknown, several studies have begun to elucidate the physiological roles of *Acer* and a closely related gene, *Ance*, either through the use of ACE-Is or genetic nulls (Figure 1B). Such studies may provide insight into how inhibition of Acer using captopril or a null mutation, suppresses AD-related phenotypes. Both genes are broadly expressed in a variety of tissues throughout development and adult stages of flies (Chintapalli et al., 2007). However, only Ance is highly expressed in male accessory glands of the fly reproductive system suggesting a role in male fertility similar to that of mammalian gACE. Accordingly, males homozygous for hypomorphic alleles of *Ance* are infertile (Hurst et al., 2003; Rylett et al., 2007). Not limited to this role, *Ance* has also been suggested to influence aging in flies. Specifically, Gabrawy et al. (2019) examined the effect of lisinopril (an ACE-I) on *Drosophila* lifespan and found that lisinopril extended life span due to inhibiting Ance. They demonstrated this by first showing that knockdown of *Ance* extended lifespan and that treatment of lisinopril failed to enhance this effect.

**Figure 2:**
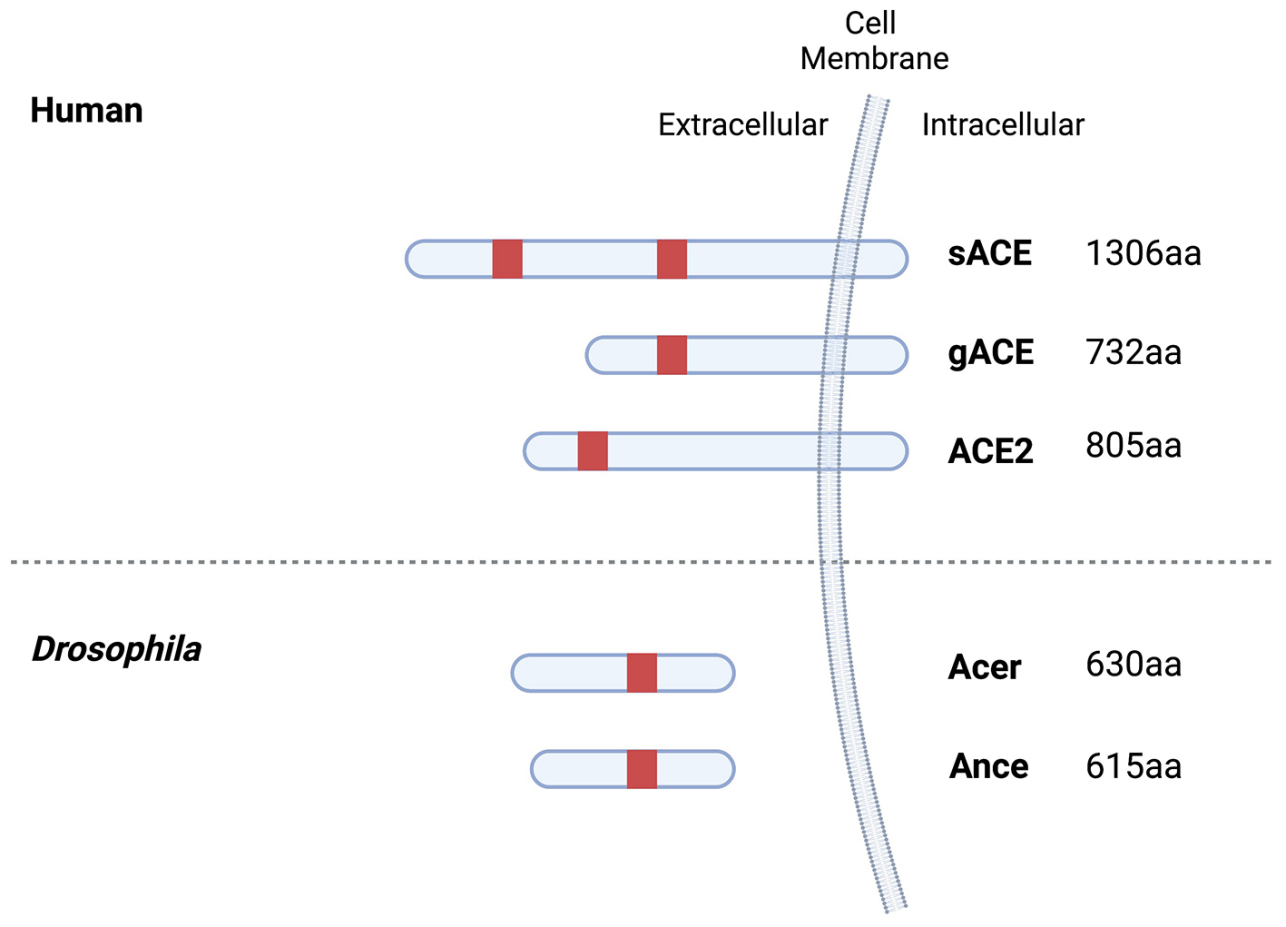
ACE family members in humans and *Drosophila*. Ance and Acer are homologous to ACE and share 61% (45% identity, 48% coverage) and 58% (41% identify, 45% coverage) amino acid similarity with ACE. Active site domains containing the conserved catalytic consensus zinc-binding motif (HEXXH) are indicated in red. sACE possess two active protein domains (N- and C- domain) whereas gACE, ACE2, Ance and Acer only have one. gACE is identical to that of the C domain of sACE except for its first 36 residues. Human ACE and ACE2 are integral-membrane proteins whereas *Drosophila* Ance and Acer lack a transmembrane domain. (Created with BioRender.com).

In contrast to *Ance*, *Acer* is highly expressed in adult heads, fat body (analogous to mammalian white adipose tissue and liver) and cardiac cells suggesting a role in different physiological processes, including cardiac function and metabolism (Crackower et al., 2002; Carhan et al., 2011). In fact, studies by Crackower et al. (2002) initially showed that an *Acer* mutation generated by a transposon (P-element) insertion gave rise to defects in heart morphogenesis that resulted in embryonic lethality. In line with these findings, Liao et al. (2013) presented a role for Acer in cardiac function by demonstrating that knock-down of *Acer*, specifically in adult heart tissue, results in heart defects such as impaired contractile properties. However, a contradictory study by Carhan et al. (2011) showed that flies homozygous for an *Acer* null mutation developed normally without significant heart defects, implying that previous heart development phenotypes observed by Crackower et al. (2002) may be attributed to a second-site mutation in the transposon-induced mutant line (Liao et al., 2013). Therefore, a role for *Acer* in *Drosophila* cardiac function remains to be established. Nevertheless, Carhan et al. (2011) identified a potential role for Acer in sleep regulation. Building upon a study that noted the cyclical expression of *Acer* in adult heads regulated by the circadian gene (*clock*) (McDonald and Rosbash, 2001; Carhan et al., 2011) hypothesized a possible role for Acer in circadian behavior. Indeed, they found *Acer* null flies exhibit a reduction in night-time sleep and greater sleep fragmentation. Moreover, this was also observed using an ACE-I, fosinopril.

Mechanisms underlying Acer’s contribution to defective sleep patterns remain unclear. Though, there is speculation that it may be due to changes in metabolic processes. *Acer* expression is strong in the fat body, a tissue with various functions, including protein and carbohydrate metabolism, lipid storage, and hormone secretion (Arrese and Soulages, 2010). Recent studies have also indicated a role for the fat body in regulating complex behaviors, including sleep (Yurgel et al., 2018). Therefore, Acer potentially possesses a functional role in metabolic processes in the fat body that, when disrupted, result in sleep defects. Beyond its prominent role in metabolism, the fat body plays an integral role in innate immune response regulation. It is responsible for the humoral response, synthesizing and secreting antimicrobial peptides into the hemolymph. While there is evidence in AD mouse studies, that ACE-Is mitigate neuroinflammatory responses that are known to contribute to AD pathology, it remains to be determined whether *Drosophila* Acer plays a similar role in immune response regulation.

Recently, a study by Glover et al. (2019) identified a potential role for Acer in metabolism including glycogen storage. Stored levels of lipids and glycogen in *Drosophila* are known to respond to dietary intake of sugar and yeast. This study found that under certain dietary conditions, *Acer* null mutant flies exhibit reduced glycogen levels compared to controls. However, just as with its role in sleep modulation, the mechanisms underlying this role for Acer are unknown. Nevertheless, it does pose an interesting avenue for further research to investigate the role of Acer in AD.

A prominent feature of AD is a significant reduction in glucose metabolism that is believed to contribute to disease progression and underlie cognitive dysfunction (Kumar et al., 2022). A decrease in metabolism is suggested to result from poor cerebral uptake of glucose into the brain. Interestingly, a study by Niccoli et al. (2016), showed that increasing glucose uptake in neurons in a *Drosophila* AD model, alleviated neurodegeneration and extended lifespan. Therefore it is worth exploring whether Acer plays a role in maintaining proper glucose metabolism in the brain of flies that could explain how its inhibition results in increased levels that in turn rescues cell death and memory phenotypes found in AD models.

## Conclusion

Looking for new strategies to treat AD is an unmet clinical need. Targeting the RAS system has great potential for AD therapeutics. While many studies in patients and animal models have shown promising beneficial effects from inhibiting this system, it remains unclear what mechanisms underlie these outcomes. The RAS is well studied for its peripheral role in regulating blood pressure, fluid and electrolytes. However, its role in organs such as the brain appears to be more complex with new components having been discovered. For this reason, *Drosophila* provides a unique opportunity to understand how ACE-Is may function in the context of AD. Given that only *ace* like factors have been identified in the fly, it is possible to study the role ACE has in AD in isolation without confounding effects from other RAS components. More so, it is evident from human studies that beneficial effects of ACE-Is arise from their ability to penetrate the BBB and act on central RAS. Therefore, their effects appear to be independent of their ability to regulate blood pressure. For that reason, using an invertebrate model such as Drosophila with an open circulatory system provides an advantage of disentangling the effects of ACE-Is from their vascular hemodynamic effects and focusing directly on their effects in the brain.

## Author contributions

JG wrote the first draft of the manuscript. JG and GB wrote sections of the manuscript. All authors contributed to the article and approved the submitted version.

## Funding

This work was supported by a grant from the Canadian Institutes of Health Research (PJT153063) to GB.

## Conflict of interest

The authors declare that the research was conducted in the absence of any commercial or financial relationships that could be construed as a potential conflict of interest.

## Publisher’s note

All claims expressed in this article are solely those of the authors and do not necessarily represent those of their affiliated organizations, or those of the publisher, the editors and the reviewers. Any product that may be evaluated in this article, or claim that may be made by its manufacturer, is not guaranteed or endorsed by the publisher.
